# 

**Published:** 1902-11-15

**Authors:** 


### NOTICE TO CORRESPONDENTS.

All MSS., Letters, Books for Review, and other matters intended for the Editor should be addressed The Editor, "The Hospital," The Hospital Building, 28 & 29 Southampton Street, Strand, W.C.

The Editor cannot undertake to return rejected MS., even when accompanied by stamped directed envelope.

**Notice to Advertisers and Subscribers.**

All Advertisements, Orders for Copies of The Hospital, and Business Communications should be addressed to **THE MANAGER, (Not to the Editor)**, The Hospital, 28 & 29 Southampton Street, Strand, London, W.C.

The Cost of Subscription, post free, is as follows.—Per week, 2½d.; per quarter, 2s. 8d.; per half-year, 5s. 3d.; per annum, 10s. 6d. Foreign:—Per annum, 15s. 2d., payable, in all cases, in advance.

**NOTICE.—Vol. XXXII. of "The Hospital" (April to September 1902) in handsome cloth binding is now on sale at the office of this Journal, price, 7s. 6d. Cases for binding the Numbers contained in this Volume 1s. 6d. Index to Vol. XXXII., 2½d., post free.**

**The Monthly Part for November is now ready. Price 6d., by post 8d.**

### BOOKS RECEIVED.

BAILLIÈRE, TINDALL AND COX.

"Anæsthetics." By J. Blumfeld, M.D. (H. K. Lewis).

"Cancer of the Uterus." By Arther Lewers, M.D., F.R.C.P.

"The Theory and Practice of Infant Feeding." By Henry Dwight Chapin, A.M., M.D.

JOHN BALE AND SONS.

"A Handbook of the Open-air Treatment and Life in an Open-air Sanatorium." Second Edition. By Dr. C. Reinhardt and Dr. David Thomson.

EYRE AND SPOTTISWOODE.

"Reports and Papers on Bubonic Plague." By Dr. R. Bruce Low.

EDWARD ARNOLD.

"Practical Physiology." By A. P. Beddard, M.D., Leonard Hill, M.B., F.R.S., J. S. Edkins, M.K.M.B., J. J. R. Macleod, M.B., and M. S. Pembrey, M.D.

### SCRAPS AND GLEANINGS.

The King has given a donation of £25 to the Royal Association in aid of the Deaf and Dumb.

The King has made a donation of 50 guineas towards the enlargement of the National Schools in connection with Windsor Parish Church.

Hospital Saturday this year was the twenty-ninth time this day has been yearly observed since the inception of the movement. The sum of £325,053 has been raised and distributed amongst the medical charities.

The committee which has been formed with the object of raising funds to found and endow rent-free cottages for deserving men of the Bedfordshire Regiment is now hard at work. A cottage has been presented to the regiment by Mr. Debenham, and a sum of £300 is required for investment to meet the expenses of yearly repairs, rates and taxes, etc., in perpetuity, towards which £94 has been collected. The inmates of the cottages will be selected by the regiment, and preference will be given to the men disabled by the South African War. Contributions may be sent to Messrs. Barnard & Co., the Bank, Bedford, for the account of the Bedfordshire Regiment Cottage Homes Fund.

The directors of the Ecclesiastical Insurance Office have given to the Queen Victoria Clergy Fund a sum of £500, one moiety of which is to be applied as income of the central fund, and the other moiety for the endowment of benefices.

The Merchant Seamen's Orphan Asylum at Snaresbrook is in future to be called the Royal Merchant Seamen's Orphan Asylum. This institution was founded in 1827.

On behalf of the Irish committee of the Women's Memorial to Queen Victoria, Sir John Arnott and Colonel Lindsay have forwarded £5,858 to the Marchioness of Londonderry, president of the central committee. This amount will be invested and handed to the trustees of Queen Victoria's Jubilee Institute for Nurses for the benefit of the work of the Queen's nurses in Ireland.

### The Student of Medicine.

### APPOINTMENTS.

**S. C. Allen,** M.B., B.Sc., appointed Second Assistant Medical Officer to the Seacliff Lunatic Asylum, New Zealand. **Wilfred Allport,** M.B., B.S.Lond., M.R.C.S., L.R.C.P., appointed Honorary Ophthalmic Surgeon to the Stratford-on-Avon Hospital. **M. C. B. Anderson,** M.R.C.S, L.R.C.P.Lond., appointed District Medical Officer of the Weymouth Union. **H. A. L. Banham,** L.R.C.P., L.R.C.S.Edin., appointed Medical Officer of Health to the Worsborough Urban District Council. **John Biggam,** M.D.Edin., appointed District Medical Officer of the Dudley Union. **W. Billington,** M.B., B.S.Lond., F.R.C.S.Eng., appointed Surgeon to Out-patients to the Queen's Hospital, Birmingham. **G. H. S. Blackburne,** M.B., Ch.B.Melb, D.P.H., appointed Medical Officer and Inspector for the Prevention of Tuberculosis in Perth, Western Australia. **H. C. Bristowe,** M.D.Lond., appointed Medical Officer and Public Vaccinator for the Wrington District of the Axbridge Union. **Reginald Davies,** M.B., Ch.M.Syd., appointed Resident Medical Officer to the North-Eastern Hospital for Children, Hackney Road. **S. L. Dawkins,** M.B., Ch.B.Edin., appointed Health Officer to the Local Board of Health for the Mudla Wirra North, South Australia. **Edward Elphick,** L.R.C.P.Lond., M.R.C.S.Eng., appointed Health Officer to the Newcastle Local Board, Western Australia. **J. C. Galloway,** M.B., Ch.B., appointed Medical Officer and Dispenser to the Northern Infirmary, Inverness. **Malcolm Gillies,** M.B., M.S.Glas., appointed Medical Officer at Bowen, and Health Officer for the Port of Bowen, Queensland. **Henry John Hewer,** M.B., appointed Visiting Surgeon to the Gaol at Blackall, Queensland. **Harry E. Hewitt,** M.D., B.S.Lond., D.P.H.Camb., appointed Assistant Pathologist and Assistant Curator of the Museum to the Westminster Hospital. **H. T. Kelsall,** M.D., Ch.B.Lond., appointed Health Officer for South Perth, Western Australia. **Lawson Tait McClintock,** M.B., Ch.B.Edin., appointed Medical Officer for the Workhouse and No. 2 District of the Loddon and Clavering Union. **A. G. McGowan,** M.B Melb., appointed Medical Officer of the Benevolent Asylum, Ballarat, Victoria. **W. J. Maguire,** M.B., B.Ch., R.U.I., appointed Surgeon to the North District of the Royal Irish Constabulary in Belfast. **Gordon William S. Marr,** M.B.Syd., appointed Assistant Medical Superintendent at the Hospital for Insane, Goodna, Queensland. **A. F. Messiter,** M.R.C.S., L.R.C.P.Lond., appointed Certifying Factory Surgeon for the Epworth District of the County of Lincoln. **P. D. Minchin,** L.R.C.P., L.R.C.S.Edin., appointed District Medical Officer of the Guildford Union. **Harold F. Mole,** L.R.C.S.Eng., appointed Honorary Assistant Surgeon to the Bristol Royal Infirmary. **J. Gawler Murray,** L.R.C.P.&S.Edin., L.F.P.S.G., appointed Admiralty Surgeon and Agent at Scarborough. **S. T. Plumbe,** M.D.Lond., M.R.C.S.Eng., appointed Certifying Factory Surgeon for the Maidenhead District of the County of Berks, and the Taplow District of the County of Bucks. **William Rose,** M.B., F.R.C.S., appointed Emeritus Professor of King's College, and Consulting Surgeon to King's College Hospital. **Linford E. Row,** M.D.Brux., L.R.C.P.&-S.Edin., appointed Medical Superintendent at the Hospital for the Insane, Goodna, Queensland. **H. H. Rubra,** M.D.Brux., M.R.C.S.Eng., L.R.C.P.Lond., appointed Anæsthetist to the Great Northern Central Hospital. **Mrs. Scharlieb,** M.D., M.S., appointed Physician for Diseases of Women to the Royal Free Hospital, Gray's Inn Road. **J. Goodall Strickland,** M.B.Lond., etc., appointed Lecturer on Biology in the Medical School, Middlesex Hospital. **Miss Ethel Vaughan,** M.D., B.S., appointed Assistant Physician for Diseases of Women to the Royal Free Hospital, Gray's Inn Road. **Hedley C. Visick,** M.R.C.S., L.R.C.P., appointed Assistant Anæsthetist to the Great Northern Central Hospital, London.

### "The Hospital" Institutional Library.

"Hospitals and Asylums of the World." 4 Vols., with a Portfolio of Plans, complete £12 12s.

"Burdett's Hospitals and Charities, 1902." 5s.

"Cottage Hospitals, General, Fever, and Convalescent." 10s. 6d.

"Hospital Expenditure: The Commissariat." 2s. 6d.

"A Legal Handbook for the Use of Hospital Authorities." 2s. 6d.

"The Cottage Hospital Case Book or Register of Patients." 10s. and 12s. 6d.

"The Furnishing and Appliances of a Cottage Hospital." 6d.

All these are published by the Scientific Press, Ltd., and may be obtained through any bookseller, or direct from the publishers, 28 and 29 Southampton Street, Strand, London, W.C.

